# The STAR RNA binding proteins GLD-1, QKI, SAM68 and SLM-2 bind bipartite RNA motifs

**DOI:** 10.1186/1471-2199-10-47

**Published:** 2009-05-20

**Authors:** André Galarneau, Stéphane Richard

**Affiliations:** 1 Terry Fox Molecular Oncology Group and the Bloomfield Center for Research on Aging, Lady Davis Institute for Medical Research, Sir Mortimer B. Davis Jewish General Hospital, and Departments of Oncology and Medicine, McGill University, Montréal, Québec, Canada, H3T 1E2; 2 Current address: Schering-Plough Canada Inc, Kirkland QC, H9H 4M7; 3 Segal Cancer Centre, 3755 Côte Ste-Catherine Road, Montréal, Québec, Canada H3T 1E2

## Abstract

**Background:**

SAM68, SAM68-like mammalian protein 1 (SLM-1) and 2 (SLM-2) are members of the K homology (KH) and STAR (signal transduction activator of RNA metabolism) protein family. The function of these RNA binding proteins has been difficult to elucidate mainly because of lack of genetic data providing insights about their physiological RNA targets. In comparison, genetic studies in mice and *C. elegans *have provided evidence as to the physiological mRNA targets of QUAKING and GLD-1 proteins, two other members of the STAR protein family. The GLD-1 binding site is defined as a hexanucleotide sequence (NACUCA) that is found in many, but not all, physiological GLD-1 mRNA targets. Previously by using Systematic Evolution of Ligands by EXponential enrichment (SELEX), we defined the QUAKING binding site as a hexanucleotide sequence with an additional half-site (UAAY). This sequence was identified in QKI mRNA targets including the mRNAs for myelin basic proteins.

**Results:**

Herein we report using SELEX the identification of the SLM-2 RNA binding site as direct U(U/A)AA repeats. The bipartite nature of the consensus sequence was essential for SLM-2 high affinity RNA binding. The identification of a bipartite mRNA binding site for QKI and now SLM-2 prompted us to determine whether SAM68 and GLD-1 also bind bipartite direct repeats. Indeed SAM68 bound the SLM-2 consensus and required both U(U/A)AA motifs. We also confirmed that GLD-1 also binds a bipartite RNA sequence *in vitro *with a short RNA sequence from its *tra-2 *physiological mRNA target.

**Conclusion:**

These data demonstrate that the STAR proteins QKI, GLD-1, SAM68 and SLM-2 recognize RNA with direct repeats as bipartite motifs. This information should help identify binding sites within physiological RNA targets.

## Background

The K homology (KH domain) is a prevalent RNA binding domain that is an evolutionarily conserved domain initially identified as a repeated sequence in the heteronuclear ribonucleoprotein particle (hnRNP) K [1]. The KH domain is a small protein module consisting of 70 to 100 amino acids and it is the second most prevalent RNA binding domain next to the RRM (RNA recognition motif) [2]. The RNA binding property of the KH domain was initially shown for FMRP, the gene product of the human fragile X syndrome and hnRNP K [3]. The KH domain is often found in multiple copies within proteins (15 in vigilin) and there is a subfamily that contains a single copy KH domain that is larger referred to as a maxi-KH domain [4].

The KH domain makes direct protein-RNA interactions with a three-dimensional β_1_α_1_α_2_β_2_β_3 _topology with an additional C-terminal α helix (α_3_) for maxi-KH domains [1]. The feature of KH domains is an invariant GXXG loop located between α_1_/α_2 _that provides close contact with the phosphate groups such that the neighboring nucleotides can form Watson and Crick base pairing with conserved amino acids within the KH domain [5,6]. The structure determination of the KH domains has also been solved with single-stranded DNA, demonstrating that certain KH domains may accommodate either RNA or ssDNA within their active site [1,7-9].

There exists a subfamily of KH domains that contain extended loops between β1/α1 and β2/β3 and that contain an additional C-terminal helix in their topography [10]. These maxi-KH domain proteins contain conserved sequences immediately at the N- and C-terminal of the KH domain. The entire region is referred to as the STAR/GSG (signal transduction activator of RNA metabolism/GRP33, SAM68, GLD-1) domain [4,11,12]. Although STAR proteins contain single KH domains, dimerization is required for RNA binding [13]. The STAR proteins are mammalian Sam68, SLM-1, SLM-2, QKI, SF1, *C. elegans *GLD-1, *Drosophila *How, KEP1, Sam50 and *Artemia Salina *GRP33 [4]. STAR proteins have been shown to function in pre-mRNA splicing [14-18], mRNA export [19-21], mRNA stability [22,23] and protein translation [24-28]. Genetic evidence has implicated the STAR RNA binding proteins in many cellular processes. These include the role of the QKI isoforms in the process of myelination of the central nervous system [29], GLD-1 in the germline determination [30-32], How in muscle and tendon differentiation [4], Kep1 in cell death processes [33] and Sam68 in bone marrow mesenchymal cell fate [34] and motor defects [35]. Genetic data has also implicated simple KH domain proteins FMRP in mental retardation and Nova in paraneoplastic neurologic disorders [2].

SF1 or branch point binding protein (BBP) was shown to recognize the branchpoint site RNA sequence (UACUAAC) [6,36] and structure determination has shown that there is direct protein-RNA contact [6]. These studies have provided necessary information about the contact sites of maxi-KH domains and their similarities/differences with simple KH domains proteins such as Nova. Based on this information, Ryder and coworkers showed that GLD-1 binds a hexanucleotide sequence (NACUCA) and proposed it as the STAR binding site [37]. In a previous effort by using Systematic Evolution of Ligands by EXponential enrichment (SELEX) [38], we defined the QKI RNA binding consensus sequence to be a bipartite motif consisting of a core NACUAAY (where Y is a pyrimidine) sequence with an neighboring half-site (UAAY) [39]. In the present study, we define for the first time the RNA binding specificity of the mammalian STAR protein, SLM-2. We identified using SELEX the SLM-2 consensus sequence as a direct U(U/A)AA repeat. The bipartite nature of the consensus RNA sequence was essential for high affinity RNA binding activity to SLM-2. The identification of a bipartite mRNA binding site for QKI [39] and now for SLM-2 prompted us to further determine whether SAM68 and GLD-1 also bound bipartite direct repeats. Indeed SAM68 and GLD-1 required bipartite RNAs, demonstrating that the STAR proteins SLM-2, SAM68, QKI and GLD-1 bind direct RNA repeats as a bipartite motif in target RNAs.

## Results

### The identification of the SLM-2 RNA binding site by using SELEX

**Figure 1:**
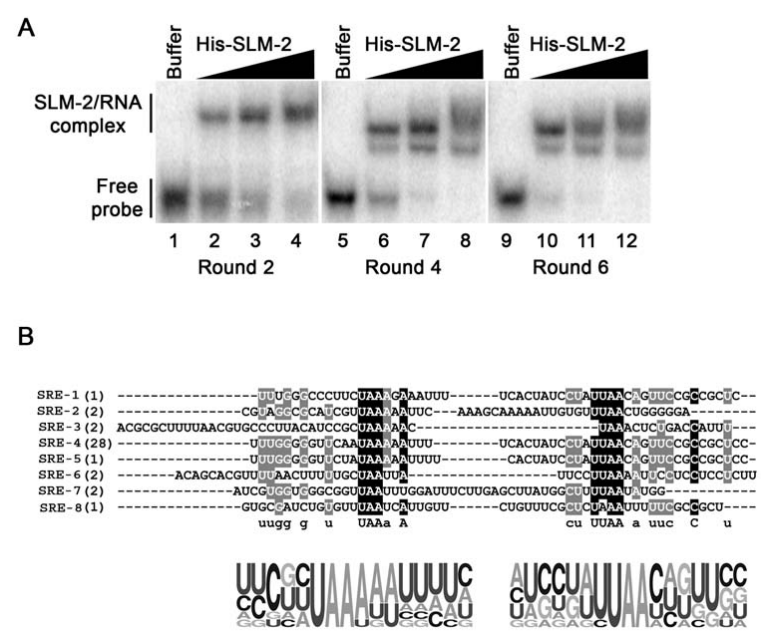
**SLM-2 RNA ligands identified**. (*A*) EMSAs of pooled RNAs identified in rounds 2, 4 and 6 using increasing concentrations of His-SLM-2. The protein/RNA complex was separated from the free probe on a native PAGE. The migration patterns of unbound RNAs (free probe) and protein bound RNAs (SLM-2/RNA complex) are indicated on the left. (*B*) The sequences of 8 unique RNAs bound to SLM-2 after six cycles of SELEX. Both identified motifs are aligned and black undermark. Illustrated, underneath the sequences is the probability matrix (graphic logo) based on all the 8 different sequences, depicting the relative frequency of each residue at each position within the selected motif.

To identify the binding motif for the SLM-2 RNA binding protein, we performed SELEX to enrich for high affinity RNA ligands. Bacterial recombinant SLM-2 expressed as a histidine epitope tagged fusion protein was generated and purified for the assay. Synthetic RNAs were transcribed with the T7 RNA polymerase from DNA pools of 52-nucleotide random-mers estimated at a complexity of 1.0 × 10^14 ^and we randomly sequenced 20 RNA molecules from the initial library and noted, as expected, that each sequence was unique [39]. The transcribed RNAs were generated in the presence of ^32^P-α-UTP such that the amount of specific SLM-2 bound RNAs could be measured after each round. After six cycles of selection, we observed an approximately 10% of binding of the initial input (not shown), demonstrating that we indeed had enriched specific sequences. To confirm the SELEX amplification of the SLM-2 specific RNA ligands, we performed a gel electromobility shift assays (EMSA) with purified pools of RNA transcripts isolated from rounds 2, 4 and 6. The RNAs were ^32^P-labelled and incubated with buffer or increasing concentration of His-SLM-2. The SLM-2/RNA complexes were observed as slow migrating complexes on native gel electrophoresis (Fig. 1A). More efficient RNA binding was observed in round 6 than rounds 2 and 4 (compare the free probe remaining from lanes 2 and 6 with lane 10). After round 6, the SLM-2 bound RNAs were converted into cDNAs, subcloned and sequenced. The sequence of 43 clones revealed that 11 clones were unique (Table 1). The clones were referred to as SLM-2 response element (SRE)-1 to 11. Class I RNAs contained a bipartite motif consisting of direct repeats of the sequence U(U/A)AA (Table 1). Our data show that the selected RNA aptamers contained a bipartite motif with direct repeats and the spacing between the repeats varied from 3 (SRE-3) to 25 (SRE-7) nucleotides (Table 1 and Fig. 1B). The 3 RNAs identified that did not contain the bipartite sequence (SRE-9, -10, -11) were grouped in Class II and since ~10% of RNAs from round 6 bound SLM-2, Class II RNAs are likely to represent non-binders. No apparent secondary structure was identified in the SREs using the prediction of RNA secondary structure program MFOLD (data not shown). Taken together, we have identified a bipartite motif consisting of direct repeats of the sequence U(U/A)AA as the SLM-2 RNA binding site.

**Table 1:** Selected SLM-2 bound RNA ligands

**Ligand**	**Sequence**	***n***
**Class I**		
SRE-1 (1)	UUUGGGCCCUUC**UAAA**GAAAUUUUCACUAUCCUA**UUAA**CAGUUCCGCCGCUC	1
SRE-2 (2)	CGUAGGCGCAUCG**UUAAA**AAUUCAAAGCAAAAAUUGUGU**UUAA**CUGGGGGA-	2
SRE-3 (2)	ACGCGCUU**UUAA**CGUGCCCUUACAUCCGC**UAAA**AAC**UAAA**CUCUGACCAUUU	2
SRE-4 (28)	UUUGGGGGUUCAA**UAAA**AAUUUUCACUAUCCUA**UUAA**CAGUUCCGCCGCUCC	28
SRE-5 (1)	UUUGGGGGUUCUA**UAAA**AAUUUUCACUAUCCUA**UUAA**CAGUUCCGCCGCUCC	1
SRE-6 (2)	ACAGCACGUU**UUAA**CUUUUUGCUAAUUAUUCCU**UAAA**AUUCCUCCUCCUCUU	2
SRE-7 (2)	AUCGUGGUGGGCGG**UUAA**UUUGGAUUUCUUGAGCUUAUGGCUU**UUAA**UAUGG	2
SRE-8 (1)	GUGCGAUCUGUGU**UUAA**UCAUUGUUCUGUUUCGCUC**UAAA**UUUUUCGCCGCU	1
**Class II**		
SRE-9 (2)	GCGGUUACGGGAUCCAUGUAGACGCACAUAUUAUAUGGGAUUAGGUAGACUG	2
SRE-10 (1)	GCUGGGGGUUGAUCCACUAUUUCCACAGCGGCAGCAACAGUUCCGCCACUUC	1
SRE-11 (1)	AUCGGGGGGGGCGG**UUAA**UUUGGACUACCCGAGCAUCAGGUCCUCCGCUGGG	1

The UAAA and UUAA conserved motifs are shown in bold. *n *= number of times identified.

### A direct repeat of U(U/A)AA defines the SLM-2 RNA binding consensus sequence

**Figure 2:**
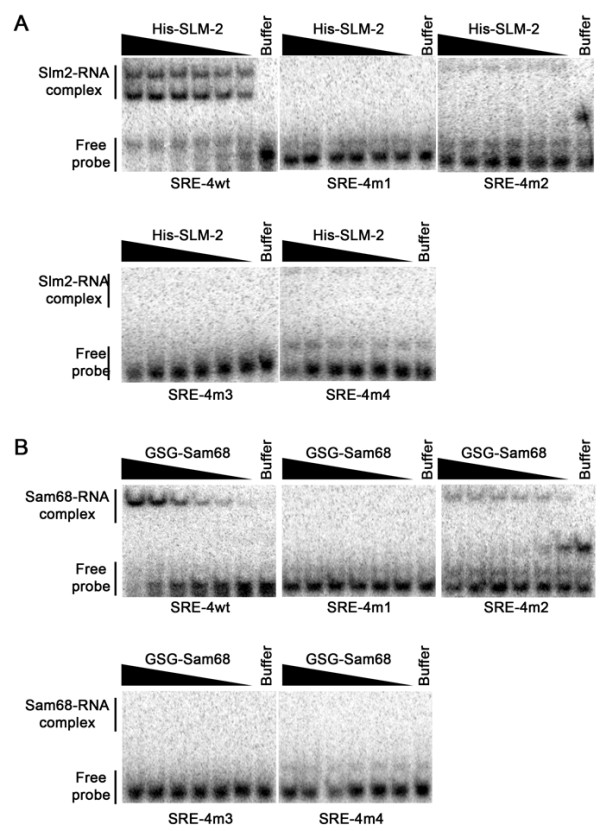
**Defining the SLM-2 response element as a bipartite RNA sequence**. EMSAs with the selected SRE-4 with decreasing concentrations of recombinant His-SLM-2 (*A*) and the SAM68 GSG domain (*B*) (by a factor of 2 from 1 μM) or with buffer alone. The RNA sequence and mutants (m1-m4) used in the reaction are shown in Table 2. Migration patterns of unbound RNAs (free probe) and protein bound RNAs (protein-RNA complex) are indicated on the left.

To define the characteristics of the SLM-2 RNA binding motif, we performed RNA binding assays with SRE-4 and SRE-7. We chose SRE-4 and -7 for further analysis because SRE-4 was the most frequently[40] identified RNA and SRE-7 contains a guanine-rich sequence at its 5'end in addition to the UUAA repeats. The 52 mer identified for SRE-4 was trimmed to a 38 mer conserving 7 nucleotides on the 5' and 3' end of the U(U/A)AA consensus sequence and designated this as SRE-4wt. This synthetic RNA bound SLM-2 with a high affinity dissociation constant of ~16 nM (Fig. 2A and Table 2) like the 52 mer SRE-4 sequences (not shown). The substitution of the either or both U(U/A)AA motifs with CCCC abolished SLM-2 binding (Fig. 2A and Table 2, SRE-4m1, m2, m3). Similarly, the replacement of the UAAA with UACC abolished RNA binding (SRE-4m4). These finding demonstrate that both tetra-nucleotide motifs (U(U/A)AA) are required for SLM-2 high affinity RNA binding.

**Table 2:** Binding affinity of SLM-2 for selected and mutated RNA ligands

**Ligand**	**Sequence**	**Binding affinity**
SRE-4wt	GGUUCUA**UAAA**AAUUUUCACUAUCCUA**UUAA**CAGUUCC	**16.3 +++**
SRE-4m1	GGUUCUAccccAAUUUUCACUAUCCUAccccCAGUUCC	**>1000 -**
SRE-4m2	GGUUCUAccccAAUUUUCACUAUCCUA**UUAA**CAGUUCC	**>1000 -**
SRE-4m3	GGUUCUA**UAAA**AAUUUUCACUAUCCUAccccCAGUUCC	**>1000 -**
SRE-4m4	GGUUCUA**UA**cccccUUUCACUAUCCUA**UUAA**CAGUUCC	**>1000 -**

SRE-7wt	AUCGUGGUGGGCGG**UUAA**UUUGGAUUUCUUGAGCUUAUGGCUU**UUAA**UAUGGG	**30.3 +++**
SRE-7m1	AUCUUguucucCGC**UUAA**UUUGGAUUUCUUGAGCUUAUGGCUU**UUAA**UAUGGG	**45.2 +++**
SRE-7m2	AUCGUaauaaaCaa**UUAA**UUUGGAUUUCUUGAGCUUAUGGCUU**UUAA**UAUGGG	**14.5 +++**
SRE-7m3	AUCGUGGUGGGCGG**UUAA**UUUGGAUUUCUUGAGCUUAUGGCUcgacgCAUGGG	**>1000 -**
SRE-7d1	AUCGUGGUGGGCGG**UUAA**UUUGGAUUU	**>1000 -**
SRE-7d2	GUGGGCGG**UUAA**UUUGGAUUUCUUGAG	**>1000 -**
SRE-7d3	GG**UUAA**UUUGGAUUUCUUGAGCUUAUG	**>1000 -**
SRE-7d4	UUUGGAUUUCUUGAGCUUAUGGCUU**UU**	**>1000 -**
SRE-7d5	UUUCUUGAGCUUAUGGCUU**UUAA**UAUGG	**>1000 -**
SRE-7d6	GGAUUUCUUGAGCUUAUGGCUU**UUAA**U	**>1000 -**
SRE-7d7	**UAA**UUUGGAUUUCUUGAGCUUAUGGCU	**>1000 -**
SRE-7d8	GG**UUAA**UUUGGAUUUCUUGAGCUUAUGGCUU**UU**	**>1000 -**
SRE-7d9	GG**UUAA**UUUGGAUUUCUUGAGCUUAUGGCUU**UUAA**UAUGG	**22.3 +++**
SRE-7d9m1	GG**UU**ccccUGGAUUUCUUGAGCUUAUGGCUU**U**ccccAUGG	**>1000 -**
SRE-7d9m2	GG**UU**ccUUUGGAUUUCUUGAGCUUAUGGCUU**UUAA**UAUGG	**≅400 +**
SRE-7d9m3	GG**UUAA**UCUGGAUUUCUUGAGCUUAUGGCUU**UU**ccUAUGG	**≅ 400 +**

U(U/A)AA motifs are shown in bold and mutated residues are small letters

We analyzed SRE-7 and identified a G-rich sequence that may represent a G quartet. We first proceeded by replacing the G-rich nucleotides with U-rich sequence and this had little effect on SLM-2 RNA binding activity (compare SRE-7m1 and SRE-7wt; Table 2). Interestingly, the replacement of the G-rich sequences with AU-rich sequences such as to introduce a third U(U/A)AA motif enhanced SLM-2 RNA binding to this RNA species (SRE-7m2; Table 2). The substitution of the downstream uUUAAu sequence with CGACGC abolished SLM-2 RNA binding consistent with the U(U/A)AA requirement (SRE-7m2). Numerous 5' and 3' deletions were performed and a minimal sequence of 40 nucleotides was identified containing both U(U/A)AA motifs that bound with a Kd of ~22.3 nM (SRE-7d9; Table 2). The substitution of the 5' or 3' U(U/A)AA motifs reduced the SLM-2 high affinity binding site (Table 2; SRE-7d9m2, m3), demonstrating that indeed SLM-2 binds RNA with high-affinity to direct repeats of U(U/A)AA.

### SAM68 binds the SLM-2 response element

SELEX has been performed with recombinant SAM68 and a UAAA consensus was defined as a necessary RNA binding site [41]. As there is 69% sequence identity between the SLM-2 and SAM68 STAR/GSG domains [42], we tested the possibility that the SLM-2 consensus (SRE-4) may be bound by Sam68. Using EMSA with recombinant Sam68 containing only the STAR/GSG domain, we observed that indeed the GSG domain of SAM68 bound the SRE-4wt RNA aptamer, but not the variants that contain mutated U(U/A)AA motifs (Fig. 2B). There was one variant of SRE-4 (SRE-4m2) that retained some binding and this is likely due to the polyuridine stretch (UUUU) that remained between the two U(U/A)AA motifs (Table 2). These findings demonstrate that SAM68 also has the capabilities to bind a bipartite U(U/A)AA consensus.

### Defining the bipartite nature of the QKI response element within the mRNAs of myelin basic protein

**Figure 3:**
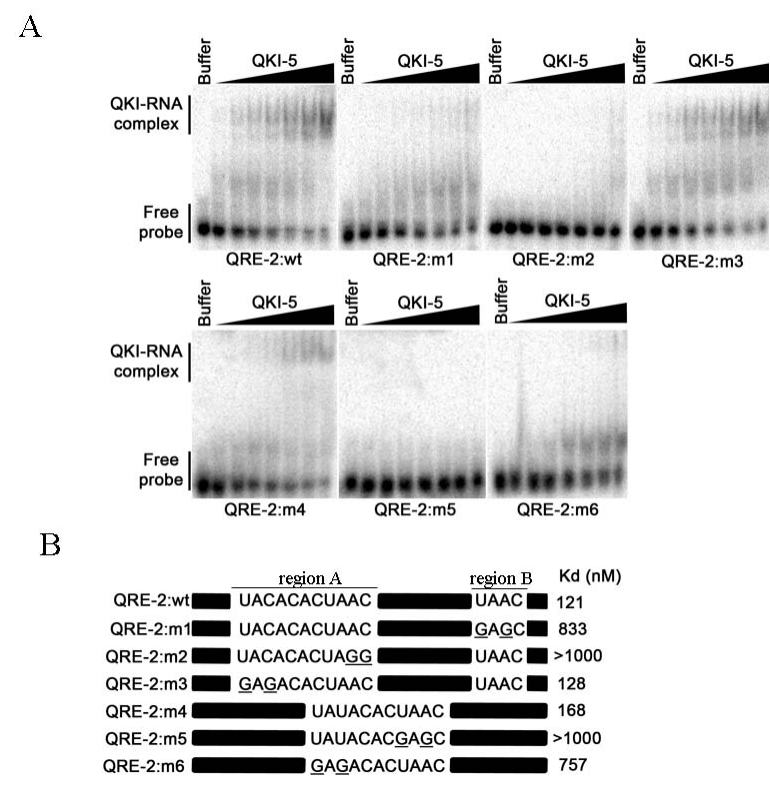
**Defining the high affinity QRE within the MBPmRNA**. (*A*) EMSAs of selected RNAs with increasing concentrations of recombinant GST-QKI-5 (by a factor of 2 from 2 nM) or with buffer alone. The RNAs used for the EMSAs are the MBP QRE-2 and variation mutants of each (m1-m6). Migration patterns of unbound RNAs (free probe) and QKI-5 bound RNAs (QKI-RNA complex) are indicated on the left. (*B*) RNA species tested in (*A*) are shown. The black bars denote sequences that are unaltered between the wild-type and the mutant versions.

The mRNAs encoding the myelin basic proteins (MBP) are known QKI targets [19,43]. The QKI RNA binding site was defined to be a core (NACUAAC) with a neighboring half-site (UAAY) [39]. The MBP QREs were defined as QRE-1 and QRE-2 [39,44]. QRE-2 is interesting as it contains two regions with an overlapping imperfect core (underlined) and half-site (bold) (UACACAC**UAAC**, QRE-2:wt) as well as downstream perfect half-site (UAAC) (Fig. 3). Alternatively, region A is recognized as the imperfect half-site (bold) (**UACA**CACUAAC) and as the perfect core (underlined). To define the requirements of QRE-2, we performed EMSA with various combinations of region A and B. The QRE-2 sequences with regions A and B bound QKI with high affinity (QRE-2:wt, Kd ~121 nM) and the substitution of the UAAC half-site in region A or region B diminished considerably the RNA binding affinity (Fig. 3, QRE-2:m1, m2). The substitution of the UACA to GAGA in region A bound with high affinity demonstrating that region A supplies the perfect core (CACUAGG) and region B supplies the half-site (UAAC) of the bipartite motif. These findings demonstrated that region A without region was unable to serve as a high affinity site for QKI (QRE-2:m1). Ryder and Williamson showed that region A alone was bound with high affinity by QKI. We next centered region A and this considerably improved QKI binding with a Kd of ~168 nM (Fig. 3, QRE-2:m4). The substitution of either the UAUA to GAGA (QRE-2:m5) or the UAAC to GAGC (QRE-2:m6) significantly reduced QKI RNA binding (Fig. 3B). These findings define the QRE-2 as requiring a bipartite motif located in region A or in region A plus region B.

### GLD-1 binds a bipartite RNA motif containing the hexanucleotide

**Figure 4:**
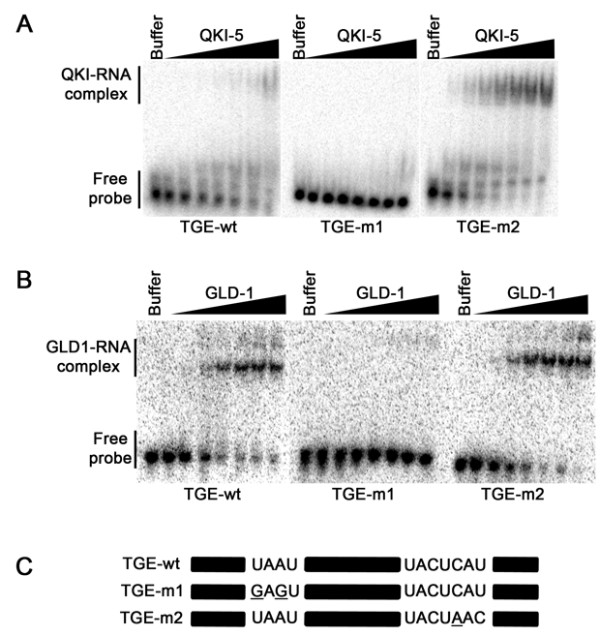
**GLD-1 binding to the *tra2*/gli element analysis**. (*A*) RNA species tested in (*B*) and (*C*) are shown. The black bars denote sequences that are unaltered between the wild-type and the mutant versions. EMSAs of the tra2/Gli element with increasing concentrations of GLD-1 (*B*) and QKI (*C*) (by a factor of 2 from 2 nM) or with buffer alone. Migration patterns of unbound RNAs (free probe) and protein/bound RNAs (GLD-1/RNA or QKI/RNA complexes) are indicated on the left.

A high affinity RNA binding site has been defined for *C. elegans *GLD-1 that consists of a hexanucleotide (NACU(C/A)A) [37]. To examine whether the GLD-1 hexanucleotide sequence also requires a similar half-site, we performed EMSA assays with a segment of the *tra2 *and *gli *repeated element (TGE) containing the hexanucleotide (UACUCAU) and its neighboring half site (UAAU)(Fig. 4A, TGE-wt). GLD-1 bound this wild-type TGE sequence and a variation of it (TGE-m2) with approximate Kd ~104 nM, defining a short sequence for GLD-1 high affinity binding (Fig. 4B). These data are consistent with previous competition experiments that defined the GLD-1 Kd ~10 nM that defined the hexanucleotide as (UACU(C/A)A) [37]. The nucleotide substitution of the half-site (UAAU to GAGU) abolished RNA binding (Fig. 4B, TGE-m1), consistent with the need for a half-site in addition to the hexanucleotide. Similar binding experiments were performed with QKI and we observed that TGE-m2 is essentially a QRE bound with high affinity, whereas the wild-type TGE bound with a moderate affinity of approximately 300 nM (Fig. 4C). The TGE-m1 was not bound by QKI (Fig. 4C). In summary, these data identify the GLD-1 RNA binding motif as bipartite as observed with SLM-2, QKI, and Sam68.

## Discussion & Conclusion

In the present study, we identified a SLM-2 consensus sequence as direct U(U/A)AA repeats using SELEX. The bipartite nature of the consensus sequence was essential for SLM-2 high affinity RNA binding. The identification of a bipartite mRNA binding site for QKI [39] and now SLM-2 prompted us to determine whether SAM68 and GLD-1 also bound bipartite direct repeats. Indeed SAM68 bound the SLM-2 consensus and required both U(U/A)AA motifs. Also, GLD-1 required sequences within the UAAY half-site in addition to its conservative consensus NACU(C/A)A, defining a GLD-1 bipartite motif. Taken together, these data demonstrate that the STAR proteins SLM-2, SAM68, QKI and GLD-1 bind direct RNA repeats.

### Defining the SLM-2 RNA binding site as U(U/A)AA repeats

We identified SLM-2 in 1999 by searching databases with SAM68 sequences [42]. Independently SLM-2 (called T-STAR) was identified as an interacting protein of RBM, an RNA binding protein involved in spermatogenesis [45]. SLM-2 is known to bind homopolymeric RNA [42], localize to SAM68 nuclear bodies (SNBs) [46], regulate alternative splicing [16] and dimerize with SAM68 and SLM-1 [42]. SLM-2 is post-translationally modified to contain methylarginines [47] and phosphotyrosines, the latter impairs its ability to associate with RNA [48]. The expression of SLM-2 is mainly restricted to testis and brain, but its function in these tissues remains unknown [49].

Previously we showed that SLM-2 had a preference for poly (G) rich homopolymeric RNA [42], therefore, we searched the SELEX hits for poly (G) rich sequences that could possibly resemble a G-quartet as bound by FMRP [50]. The SLM-2 selected RNA (SRE-7) contained a variation of this sequence (GGnGGGnGGnnnnnnnGG), but its deletion did not affect SLM-2 RNA binding. Therefore, we next focused on the U(U/A)AA rich repeats that resemble the consensus identified with SAM68 SELEX [41]. Indeed we mapped the SLM-2 consensus sequence to direct repeats of the U(U/A)AA sequence, defining a SLM-2 RNA binding site as a bipartite motif. This motif is too frequently found in mRNAs especially in 3'-UTR to perform a bioinformatic analysis to identify the SLM-2 mRNA targets (not shown). Thus the specificity in SLM-2 function is most likely contributed by its tissue specific expression and post-translational modifications may alter its RNA binding specificity and/or accessibility.

Sam68 is known to bind cellular RNA as well as DNA [51]. Sam68 is known to have a preference for poly (U) and poly (A) homopolymeric RNA and this association is abrogated with tyrosine phosphorylation by Src kinases and BRK [52,53]. Differential display and cDNA representation difference analysis identified 29 potential RNA binding targets of which 10 bind in a KH-dependent manner [54]. Sam68 binding sequences on hnRNP A2/B1 and β-actin mRNAs were mapped to UAAA and UUUUUU nucleotide motifs, respectively and both motifs occur within specific loop structures [54]. Sam68 has also been shown to transport unspliced HIV RNAs [20]. The knockout Sam68 mice are protected against the development of osteoporosis pointing towards an enhancement of the mesenchymal stem cell differentiation along the osteogenic rather than the adipocyte pathway [34]. The mice also have motor coordination defects [35]. The identification of Sam68 in these physiological processes will help direct the search for specific physiological mRNA targets. The work performed herein demonstrates that the STAR/GSG domain of Sam68 has similar RNA binding capabilities to SLM-2, as suggested by their 69% sequence identity within their STAR/GSG domains [42].

### QUAKING: a regulator of myelination

The *quaking viable (qk*^*v*^*) *mice represent an animal model of dysmyelination [55]. The defect is summarized as an incomplete maturation of the myelin sheath. This is due to the lack in proper oligodendrocyte differentiation, resulting in the failure to transport intracellular myelin components such as the MBP mRNAs [55]. QKI null animals have been generated, but the embryos die at ~E9.5–10.5 day, providing little information about the role of QKI in myelination [56]. By using a gain-of-function approach with ectopic expression of the QKI isoforms, we showed previously that QKI-6 and QKI-7 promote oligodendrocyte differentiation by up-regulating p27^KIP1^, confirming the role for the QKI isoforms during myelination [22]. The QKI response element was defined as a core NACUAAY [44] with a neighboring UAAY [39,44]. This led to the identification of two binding sites within the mRNAs for the MBPs [39,44]. QRE-1 contains 3' adjacent half-sites that function as a moderate affinity site. In the present study, we demonstrate that region A in QRE-2 (Fig. 4) shown previously to mediate binding [44], becomes a better site with the presence of the half site from region B (Fig. 4). Our findings show that QRE-2 within the 3'UTR of MBP mRNAs is indeed a bipartite consensus sequence with a core NACUAAY and a neighboring UAAY.

The MBP mRNAs are localized at the distal processes of oligodendrocytes in intact tissue [57]. The factors necessary for MBP mRNA localization are oligodendrocyte-specific, as transfected MBP mRNA into non-glial cells did not properly localize to the cell membrane [58]. Studies performed in living cells by microinjection have shown that the MBP mRNA forms granules, which appear dispersed in the perikaryon and are transported down the processes [59]. MBP is not the only mRNA known to be localized to the distal processes of oligodendrocytes, as myelin oligodendrocytes basic protein (MOBP), alpha-CAMKII, tau, amyloid precursor protein (APP) and others are also transported to the site of myelination [60]. Transport and localization elements have been mapped in the 3' UTR of rat and mouse MBP mRNA. A 21-nucleotide sequence named RNA transport signal (RTS) mapped at nucleotide 794 to 814 of rat MBP or nucleotide 798 to 818 of mouse MBP has been identified as a transport element [61]. This sequence is homologous to several other localized mRNAs, suggesting a general transport signal. In rat oligodendrocytes, another localization element has been mapped to nucleotides 1130 to 1473 named the RNA localization region (RLR), but the region 667 to 953 containing the RTS and QRE-1 is sufficient for localization [61]. HnRNP A2 has been shown to be one of the component which binds the RTS sequence [62], and insertion of the RTS into GFP resulted in enhanced translation [63]. The mapping of QRE-2 (UACUAAC-13nt-UAAC) constitutes another element that may be necessary for proper export of the MBP mRNA into the cytoplasm and subsequent production of the MBP at its site of synthesis. It is likely that QKI works in combination with hnRNP A2 and the other components of the RNP granule in the proper transport of the MBP mRNA, its localization and its translation.

The *C. elegans *homolog of QKI is GLD-1, a known protein translation inhibitor required for germ-line differentiation [64,65]. Many GLD-1 mRNA targets have been identified [25-28] and a conservative consensus sequence of NACU(C/A)A was defined by comparing the binding specificity with SF1 [37]. Most, but not all mRNA targets [37], contain this conversed consensus sequence. The demonstration that GLD-1 like QKI requires a neighboring half-site is consistent with the ~50% sequence identity within their STAR/GSG domains.

### Sam68/SLM-2 tetranucleotide versus QKI/GLD-1 hexanucleotide sequence requirements

**Figure 5:**
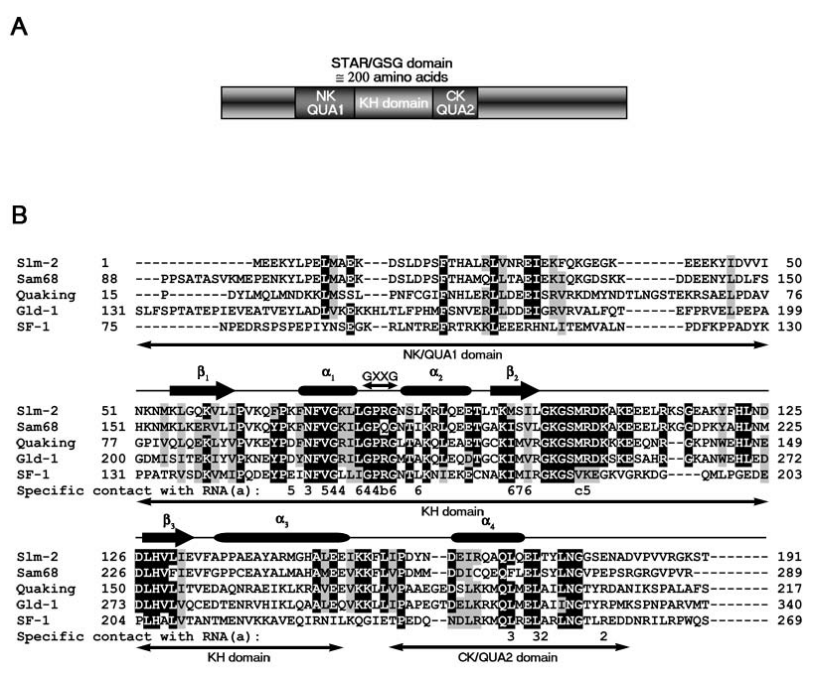
**STAR/GSG domain protein alignment**. (*A*) Diagram representing the structural and functional region of the STAR/GSG domain containing proteins. (*B*) The STAR/GSG domain of mouse SLM-2, human SAM68, mouse QKI, C. *elegans *GLD-1 and human SF-1 were aligned using ClustalW. Secondary structure, beta sheets and alpha helices, are shown on top of the sequences and region NK/QUA1, the KH domain and region CK/QUA2 are shown beneath the sequences. The critical loop between helices alpha 1 and alpha 2 with the GXXG sequence is also shown. (a) Based on [6] the RNA bases UACUAAC that contact with the specific SF-1 residues are numbered as follow U_1_A_2_C_3_U_4_A_5_A_6_C_7_. (b) Arginine 160 makes contact with U_4 _and A_6_. (c) Valine 183 makes contact with A_6 _and C_7_.

The RNA binding domain of STAR/GSG proteins consist in a maxi-KH domain flanked by two conserved sequences (Fig. 5). The NK/QUA1 and CK/QUA2 region refer to the N- and C-terminal region, respectively, flanking the KH domain. Based on the structure of the KH domain of SF-1 associated with its binding RNA molecule U_1_A_2_C_3_U_4_A_5_A_6_C_7_, the CK region makes important contacts with the RNA. All STAR domain containing proteins have the most important GXXG sequence located in a loop between the two first alpha helices of the KH domain. This sequence of residues is absolutely conserved among the STAR domain proteins and makes the contact with the RNA especially with the bases U_4_A_5_A_6_C_7_. By looking closely at the residues in the CK region that make important association with the RNA bases, we find that two residues (asterix on Fig. 5) seems to confer the SLM-2/SAM68 specificity versus the QKI/GLD-1 specificity. The SLM-2/SAM68 residues are a threonine or a serine and a conserved glutamic acid while the QKI/GLD-1/SF1 residues consist in a conserved alanine and a conserved arginine. These residues make important contact with base A_2 _which specificity is lost in the Slm-2/Sam68 consensus binding sequence. In fact, SLM-2/Sam68 binding sequence resembles in all points to the QKI/GLD-1/SF1 core binding sequence but lacking U_1_A_2_C_3 _bases.

The STAR protein SF1 structure was determined and the amino acids that contact the RNA were identified [6]. Based on these contact amino acids, it explains why SF1, QKI and GLD-1 have near identical binding specificity. The Sam68, SLM-1 and SLM-2 subfamily have different amino acids in the RNA contact position and it should be possible by amino acid substitution to convert a Sam68 domain into a GLD-1 domain that will bind the NACUA(C/A)C GLD-1 consensus sequence. Lehmann-Blount & Williamson (2005) have performed such experiments and were unable by mutagenesis to identify an amino acid 'code' that would dictate GLD-1-like versus Sam68-like specificity [66]. This led them to propose that Sam68 and hence SLM-1 and SLM-2 might not be RNA binding proteins or possess an RNA binding specificity that is fundamentally unlike that of GLD-1 [66]. The identification of a high affinity RNA target for SLM-2 with the characteristics of a GLD-1/QKI bipartite motif, demonstrates that Sam68, SLM-1 and SLM-2 subfamily are indeed RNA binding proteins, but does not exclude the possibility that they may also bind ssDNA. The challenge ahead will be to identify the physiological RNA targets linking with the phenotypes observed in mammals.

## Methods

### SELEX assay

Systematic Evolution of Ligand by EXpornential enrichement (SELEX) was performed as previously described [67]. Essentially, an oligonucleotides harboring a 52-bp random sequence surrounded by two primer binding sites, with an estimated complexity of 1 × 10^15^, were synthesized by (Invitrogen). The oligonucleotides were amplified by PCR using corresponding forward and reverse primers as previously described [67]. After PCR amplification, the sequences of 24 random clones were determined; each clone was unique and the overall base composition whoed similarity among the clones (average composition: A, 20%; U, 30%; C, 22%; G, 28%; data not shown). A purified DNA library (1 × 10^13 ^molecules) was transcribed *in vitro *using the T7 RNA polymerase (Promega) and (α-^32^P)-UTP. RNA was purified from denaturing TBE-acrylamide gels, heated to 65°C fro 5 min, and precleared using TALON Metal Affinity Resin (BD Bioscience) to absorb non-specifically bound RNAs. Unbound RNAs were incubated in binding buffer (50 mM Tris-HCl (pH 8.0), 590 mM KCl) with the recombinant His-SLM-2 for 30 min, then with TALON Metal Affinity Resin for another 30 min. After four washes with binding buffer, the RNAs were eluted by TRIzol extraction (Invitrogen). The purified RNAs were ethanol precipitated and resuspended in water with RNase-free DNase for a 15 min reaction. The DNase reaction was quenched for 10 min at 65°C. Reverse transcriptions were performed using M-MLV reverse transcriptase (Promega) and a reverse oligonucleotide annealing to the 3' primer binding site. cDNAs were then generated by PCR amplification with the reverse oligonucleotide and the forward oligonucleotide annealing to the 5' primer binding site containing the T7 promoter. After round 6, the cDNAs were amplified with the reverse primer and a forward primer containing the *Eco*RI restriction site. The DNA fragments were digested with *Eco*RI and *Bam*HI and subcloned into pBluescript SK+ (Stratagene) for blue/white selection. Forty-three random white colonies were selected, their plasmid were purified and the SELEX sequence was identified by DNA sequencing (Genome Quebec).

### RNA preparation, purification and ElectroMobility Shift Assays (EMSAs)

RNAs were prepared by run-off *in vitro *transcription of oligonucleotides harboring a T7 binding site in the presence of ^32^P- UTP, using T7 MegaShortscript (Ambion) according to the manufacturer's protocols. RNAs were purified on TBE-acrylamide gels before use. For EMSAs, a constant concentration of ^32^P-labeled RNA (100 pmol) was incubated alone with buffer or with increasing or decreasing concentrations of the corresponding tested proteins in the following buffer: 20 mM HEPES (pH7.4), 330 mM KCl, 10 mM MgCl_2_, 0.1 mM EDTA, 0.1 mg/ml heparin and 0.01% IGEPAL CA630 (Sigma). The 30 μl reaction were incubated at room temperature for 1 h, and 3.3 μl of RNA loading dye (glycerol containing 0.25% (w/v) bromophenol blue, 0.25% (w/v) xylene cyanol) was added to each. A portion (15 μl) of each sample was separated on native Tris-glycine 8%-acrylamide gels. The gels were dried and the bound and unbound RNAs were quantified using a Storm Phosphorimager (Amersham). The fraction of bound RNA was determined and plotted using the software program Prism 3.0 (GraphPad Software).

### DNA and protein preparation

Recombinant GST-QKI-5 was described previously [39]. Maltose binding protein fused to GLD-1 was a generous gift of Min-Ho Lee (University of Albany). His-SLM-2 was prepared from subcloning the coding region from GFP-SLM-2 [42] into pQE Trisystem (Qiagen) using *Bam*H1 and *Xho*I directionnal cloning. His-GSG(SAM68) was prepared by subcloning the GSG domain of mouse Sam68 into pET-18c. Protein purification was performed as per the manufacturer's instructions.

## Authors' contributions

AG performed the experiments and analyzed the data. AG and SR conceived the experiments and wrote the manuscript.

## References

[B1] Valverde R, Edwards L, Regan L (2008). Structure and function of KH domains. FEBS J.

[B2] Lukong KE, Chang KW, Khandjian EW, Richard S (2008). RNA-binding proteins in human genetic disease. Trends Genet.

[B3] Glisovic T, Bachorik JL, Yong J, Dreyfuss G (2008). RNA-binding proteins and post-transcriptional gene regulation. FEBS Lett.

[B4] Volk T, Israeli D, Nir R, Toledano-Katchalski H (2008). Tissue development and RNA control: "HOW" is it coordinated?. Trends Genet.

[B5] Lewis HA, Musunuru K, Jensen KB, Edo C, Chen H, Darnell RB, Burley SK (2000). Sequence-specific RNA binding by a Nova KH domain: implications for paraneoplastic disease and the fragile X syndrome. Cell.

[B6] Liu Z, Luyten I, Bottomley MJ, Messias AC, S H-M, R S, Zanier K, Kramer AMS (2001). Structural basis for recognition of the intron branch site RNA by splicing factor 1. Science.

[B7] Backe PH, Messias AC, Ravelli RB, Sattler M, Cusack S (2005). X-ray crystallographic and NMR studies of the third KH domain of hnRNP K in complex with single-stranded nucleic acids. Structure.

[B8] Du Z, Lee JK, Tjhen R, Li S, Pan H, Stroud RM, James TL (2005). Crystal structure of the first KH domain of human poly(C)-binding protein-2 in complex with a C-rich strand of human telomeric DNA at 1.7 A. J Biol Chem.

[B9] Braddock DT, Baber JL, Levens D, Clore GM (2002). Molecular basis of sequence-specific single-stranded DNA recognition by KH domains: solution structure of a complex between hnRNP K KH3 and single-stranded DNA. EMBO J.

[B10] Di Fruscio M, Chen T, Bonyadi S, Lasko P, Richard S (1998). The identification of two Drosophila KH domain proteins: KEP1 and SAM are members of the Sam68 family of GSG domain proteins. J Biol Chem.

[B11] Lukong KE, Richard S (2003). Sam68, the KH domain-containing superSTAR. Biochim Biophys Acta.

[B12] Vernet C, Artzt K (1997). STAR, a gene family involved in signal transduction and activation of RNA. Trends in Genet.

[B13] Chen T, Damaj BB, Herrera C, Lasko P, Richard S (1997). Self-association of the single-KH-domain family members Sam68, GRP33, GLD-1, and Qk1: role of the KH domain. Mol Cell Biol.

[B14] Paronetto MP, Achsel T, Massiello A, Chalfant CE, Sette C (2007). The RNA-binding protein Sam68 modulates the alternative splicing of Bcl-x. J Cell Biol.

[B15] Arning S, Gruter P, Bilbe G, Kramer A (1996). Mammalian splicing factor SF1 is encoded by variant cDNAs and binds to RNA. RNA.

[B16] Stoss O, Novoyatleva T, Gencheva M, Olbrich M, Benderska N, Stamm S (2004). P59 fyn-mediated phosphorylation regulates the activity of the tissue-specific splicing factor rSLM-1. Mol Cell Neurosci.

[B17] Matter N, Herrlich P, Konig H (2002). Signal-dependent regulation of splicing via phosphorylation of Sam68. Nature.

[B18] Chawla G, Lin CH, Han A, Shiue L, Ares MJ, Black DL (2008). Sam68 regulates a set of alternatively spliced exons during neurogenesis. Mol Cell Biol.

[B19] Larocque D, Pilotte J, Chen T, Cloutier F, Massie B, Pedraza L, Couture R, Lasko P, Almazan G, Richard S (2002). Nuclear retention of MBP mRNAs in the Quaking viable mice. Neuron.

[B20] Reddy TR, Xu W, Mau JKL, Goodwin CD, Suhasini M, Tang H, Frimpong K, Rose DW, Wong-Staal F (1999). Inhibition of HIV replication by dominant negative mutants of Sam68, a functional homolog of HIV-1 Rev. Nature Medicine.

[B21] Coyle JH, Guzik BW, Bor YC, Jin L, Eisner-Smerage L, Taylor SJ, Rekosh D, Hammarskjold ML (2003). Sam68 enhances the cytoplasmic utilization of intron-containing RNA and is functionally regulated by the nuclear kinase Sik/BRK. Mol Cell Biol.

[B22] Larocque D, Galarneau A, Liu HN, Scott M, Almazan G, Richard S (2005). Protection of the p27KIP1 mRNA by quaking RNA binding proteins promotes oligodendrocyte differentiation. Nat Neurosci.

[B23] Nabel-Rosen H, Dorevitch N, Reuveny A, Volk T (1999). The balance between two isoforms of the Drosophila RNA-binding protein How controls tendon cell differentiation. Mol Cell.

[B24] Saccomanno L, Loushin C, Jan E, Punkay E, Artzt K, Goodwin EB (1999). The STAR protein QKI-6 is a translational repressor. Proc Natl Acad Sci USA.

[B25] Jan E, Motzny CK, Graves LE, Goodwin EB (1999). The STAR protein, GLD-1, is a translational regulator of sexual identity in Caenorhabditis elegans. EMBO J.

[B26] Lee MH, Schedl T (2004). Translation repression by GLD-1 protects its mRNA targets from nonsense mediated mRNA decay in C. elegans. Genes & Dev.

[B27] Schumacher B, Hanazawa M, Lee M-H, Nayak S, Volkmann K, Hofmann R, Hengartner M, Schedl T, Gartner A (2005). Translational Repression of C. elegans p53 by GLD-1 Regulates DNA Damage-Induced Apoptosis. Cell.

[B28] Lee M-H, Schedl T (2001). Identification of in vivo mRNA targets of GLD-1, a maxi-KH motif containing protein required for C. elegans germ cell development. Genes & Dev.

[B29] Larocque D, Richard S (2005). Quaking KH domain proteins as regulators of glial cell fate and differentiation. RNA Biology.

[B30] Francis R, Barton MK, Kimbel J, Schedl T (1995). Control of oogenesis, germline proliferation and sex determination by the *C. elegans *gene *gld-1*. Genetics.

[B31] Francis R, Maine E, Schedl T (1995). Gld-1 a cell-type specific tumor suppressor gene in *C. elegans*. Genetics.

[B32] Crittenden SL, Bernstein DS, Bachorik JL, Thompson BE, Gallegos M, Petcherski AG, Moulder G, Barstead R, Wickens M, Kimble J (2002). A conserved RNA-binding protein controls germline stem cells in Caenorhabditis elegans. Nature.

[B33] Di Fruscio M, Styhler S, Wikholm E, Boulanger MC, Lasko P, Richard S (2003). Kep1 interacts genetically with dredd/caspase-8, and kep1 mutants alter the balance of dredd isoforms. Proc Natl Acad Sci USA.

[B34] Richard S, Torabi N, Franco GV, Tremblay GA, Chen T, Vogel G, Morel M, Cleroux P, Forget-Richard A, Komarova S (2005). Ablation of the Sam68 RNA binding protein protects mice from age-related bone loss.

[B35] Lukong KE, Richard S (2008). Motor coordination defects in mice deficient for the Sam68 RNA-binding protein. Behav Brain Res.

[B36] Peled-Zehavi H, Berglund JA, Rosbash M, Frankel AD (2001). Recognition of RNA branch point sequences by the KH domain of splicing factor 1 (mammalian branch point binding protein) in a splicing factor complex. Mol Cell Biol.

[B37] Ryder SP, Frater LA, Abramovitz DL, Goodwin EB, Williamson JR (2004). RNA target specificity of the STAR/GSG domain post-transcriptional regulatory protein GLD-1. Nat Struct Mol Biol.

[B38] Tuerk C, Gold L (1990). Systemic evolution of ligands by expontential enrichment: RNA ligands to bacteriophage T4 DNA polymerase. Science.

[B39] Galarneau A, Richard S (2005). Target RNA motif and target mRNAs of the Quaking STAR protein. Nat Struct Mol Biol.

[B40] Boisvert FM, Hendzel MJ, Masson JY, Richard S (2005). Methylation of MRE11 regulates its nuclear compartmentalization. Cell Cycle.

[B41] Lin Q, Taylor SJ, Shalloway D (1997). Specificity and determinants of Sam68 RNA binding. J Biol Chem.

[B42] Di Fruscio M, Chen T, Richard S (1999). Two novel Sam68-like mammalian proteins SLM-1 and SLM-2: SLM-1 is a Src substrate during mitosis. Proc Natl Acad Sci USA.

[B43] Li Z, Zhang Y, Li D, Feng Y (2000). Destabilization and mislocalization of the myelin basic protein mRNAs in quaking dysmyelination lacking the Qk1 RNA-binding proteins. J Neurosci.

[B44] Ryder SP, Williamson JR (2004). Specificity of the STAR/GSG domain protein Qk1: implications for the regulation of myelination. RNA.

[B45] Venables JP, Vernet C, Chew SL, Elliot DJ, Cowmeadow RB, Wu J, Cooke HJ, Artzt K, Eperon IC (1999). T-STAR/ETOILE: a novel relative of Sam68 that interacts with an RNA-binding protein implicated in spermatogenesis. Hum Mol Genetics.

[B46] Chen T, Boisvert FM, Bazett-Jones DP, Richard S (1999). A role for the GSG domain in localizing Sam68 to novel nuclear structures in cancer cell lines. Mol Biol Cell.

[B47] Côté J, Boisvert FM, Boulanger MC, Bedford MT, Richard S (2003). Sam68 RNA binding protein is an in vivo substrate for protein arginine N-methyltransferase 1. Mol Biol Cell.

[B48] Haegebarth A, Heap D, Bie W, Derry JJ, Richard S, Tyner AL (2004). The nuclear tyrosine kinase BRK/Sik phosphorylates and inhibits the RNA-binding activities of the Sam68-like mammalian proteins SLM-1 and SLM-2. J Biol Chem.

[B49] Elliott DJ (2004). The role of potential splicing factors including RBMY, RBMX, hnRNPG-T and STAR proteins in spermatogenesis. Int J Androl.

[B50] Darnell JC, Jensen KB, Jin P, Brown V, Warren ST, Darnell RB (2001). Fragile X mental retardation protein targets G quartet mRNAs important for neuronal function. Cell.

[B51] Wong G, Muller O, Clark R, Conroy L, Moran MF, Polakis P, McCormick F (1992). Molecular cloning and nucleic acid binding properties of the GAP-associated tyrosine phosphoprotein p62. Cell.

[B52] Wang LL, Richard S, Shaw AS (1995). p62 association with RNA is regulated by tyrosine phosphorylation. J Biol Chem.

[B53] Derry JJ, Richard S, Carvajal HV, Ye X, Vasioukhin V, Cochrane AW, Chen T, Tyner AL (2000). Sik (BRK) phosphorylates Sam68 in the nucleus and negatively regulates its RNA binding activity. Mol Cell Biol.

[B54] Itoh M, Haga I, Li Q-H, Fujisawa J-I (2002). Identification of cellular mRNA targets for RNA-binding protein Sam68. Nucl Acids Res.

[B55] Chenard CA, Richard S (2008). New implications for the QUAKING RNA binding protein in human disease. J Neurosci Res.

[B56] Li Z, Takakura N, Oike Y, Imanaka T, Araki K, Suda T, Kaname T, Kondo T, Abe K, Yamamura K (2003). Defective smooth muscle development in qkI-deficient mice. Dev Growth Differ.

[B57] Verity AN, Campagnoni AT (1988). Regional expression of myelin protein genes in the developing mouse brain: In situ hybridization studies. J Neurosci Res.

[B58] Boccaccio GL, Colman DR (1995). Myelin basic protein mRNA localization and polypeptide targeting. J Neurosci Res.

[B59] Ainger K, Avossa D, Morgan F, Hill SJ, Barry C, Barbarese E, Carson JH (1993). Transport and localization of exogenous myelin basic protein mRNA microinjected into oligodendrocytes. J Cell Biol.

[B60] Kindler S, Wang H, Richter D, Tiedge H (2005). RNa transport and local control of translation. Annu Rev Cell Dev Biol.

[B61] Ainger K, Avossa D, Diana AS, Barry C, Barbarese E, Carson JH (1997). Transport and localization elements in myelin basic protein mRNA. J Cell Biol.

[B62] Hoek KS, Kidd GJ, Carson JH, Smith R (1998). hnRNP A2 selectively binds the cytoplasmic transport sequence of myelin basic protein mRNA. Biochemistry.

[B63] Kwon S, Barbarese E, Carson JH (1999). The cis-acting RNA trafficking signal from myelin basic protein mRNA and its cognate trans-acting ligand hnRNP A2 enhance cap-dependent translation. J Cell Biol.

[B64] Lee MH, Schedl T (2006). RNA-binding proteins. WormBook.

[B65] Kimble J, Crittenden SL (2007). Controls of germline stem cells, entry into meiosis, and the sperm/oocyte decision in Caenorhabditis elegans. Annu Rev Cell Dev Biol.

[B66] Lehmann-Blount KA, Williamson JR (2005). Shape-specific nucleotide binding of single-stranded RNA by the GLD-1 STAR domain. J Mol Biol.

[B67] Buckanovich RJ, Darnell RB (1997). The Neuronal RNA Binding Protein Nova-1 Recognizes Specific RNA Targets In Vitro and In Vivo. Mol Cell Biol.

